# Viral Ecology and Natural Infection Dynamics of Kaeng Khoi Virus in Cave-Dwelling Wrinkle-Lipped Free-Tailed Bats (*Chaerephon plicatus*) in Thailand

**DOI:** 10.3390/diseases9040073

**Published:** 2021-10-15

**Authors:** William A. Neill, Rebekah C. Kading

**Affiliations:** 1 School of Public Health, Johns Hopkins University, Baltimore, MD 21205, USA; neillwa43@gmail.com; 2 Department of Microbiology, Immunology, and Pathology, Colorado State University, Fort Collins, CO 80523, USA

**Keywords:** chiroptera, emerging virus, orthobunyavirus, pathology, experimental infection, reservoir, arbovirus

## Abstract

Kaeng Khoi virus (KKV; Order: *Bunyavirales*), is an endemic viral infection of the wrinkle-lipped free-tailed bat (*Chaerephon plicatus* aka *Tadarida plicata plicata*). Little is known about the ecology and maintenance of KKV within the bat population, nor the infection dynamics and transmission among bats or between bats and other vertebrates. Therefore, KKV was studied in Kaeng Khoi cave, Saraburi province, Thailand, during 1973–1974 with the objectives to (1) characterize the seasonal infection rates of KKV in the context of the bat population ecology, and (2) describe the infection dynamics and viral shedding by naturally- and experimentally-infected bats. To this end, the free-tailed bat population was estimated by a series of timed photographs taken during the evening exodus. The case population of 900,000 adult bats doubled at the time of weaning of the young and returned to its previous level soon thereafter. The newborn bats had neutralizing antibodies to KKV that were likely to be maternal in origin. The KKV antibody prevalence in adult bats was high (69–91%) in March–May and low (29–40%) in August and September. Kaeng Khoi virus was isolated from 75% of dead and 50% of moribund bats, but was not found in nearly 400 apparently healthy bats. Virus was present in saliva, urine and blood of most of the naturally-moribund bats tested. Consistent with observations from naturally-infected bats, experimental infection of bats with KKV revealed significant liver pathology, also suggestive that this is not a benign infection. Kaeng Khoi virus is an endemic, year-round infection maintained by the annual recruitment of a large number of immunologically-naïve juvenile bats. Moreover, it produces an acute infection in the bat, either leading to death by hepatitis, or immunity.

## 1. Introduction

Kaeng Khoi virus (KKV) was first isolated from a wrinkle-lipped free-tailed bat (*Chaerephon plicatus*; aka *Tadarida plicata plicata*),in Saraburi province, Thailand in 1969 [1]. The virus is a member of the order *Bunyavirales*, family *Peribunyaviridae;* it clusters phylogenetically with Mojuí dos Campos virus (MDCV) which was isolated from an unknown species of bat in Brazil in 1976 [2], and Nyando virus, which has been isolated from mosquitoes throughout Africa and causes febrile disease in humans [3,4,5]. Kaeng Khoi virus was isolated from dead free-tailed bats and arthropods collected in Kaeng Khoi cave [1,6] and has since also been isolated from bats (*Chaerephon plicatus*) in Cambodia [7] and bat flies (*Eucampsipoda sundaica*) associated with rousette bats in the Yunnan Province of China [8]. Guano miners who worked in Kaeng Khoi cave had detectable neutralizing antibodies to the virus [1], although the pathogenic potential and disease burden in the local community resulting from KKV infection remains unknown. Other than these few reports of virus isolations and phylogenetic analysis, the ecology and transmission of KKV has not been studied.

The currently-recognized genus *Chaerephon* comprises Old World free-tailed bats in the family *Molossidae* [9]. These are small (10–20 g), insectivorous bats with reddish brown to almost black coloration. Bats in this genus may be found singly, although the majority of the species are gregarious, forming large roosts, which may have from a few hundred to several million bats throughout Southeast Asia and the Indian subcontinent. The larger roosts are generally located in caves, whereas smaller roosts are in man-made structures, rock crevices and hollow trees [10]. The wrinkle-lipped free-tailed bat exhibits continuous bimodal polyestry in that there are two birthing periods per year (around April and October) in two distinct seasons of parturition [11]. In Cambodia, pregnant bats were found in February–April and August–September, and lactating females were captured in April–July and October–January [11]. Newborns are not able to fly during their suckling period, which is about six weeks in duration. The bats born in in mid-April become volant between late May and early June, at which time they join the foraging population. While it is recognized that KKV is found in these free-tailed bats, the circulation of KKV in the wild bat population from season-to-season and patterns of virus infection and shedding by different age groups of bats is not known.

The objective of this study was to understand how KKV is maintained in the bat population, and how viral transmission likely occurs among bats or between bats and other vertebrates. In the field, estimates were made of the population size, age structure, and antibody prevalence in different age cohorts of bats at different times of the year. These studies also illuminated how recruitment of immunologically-naïve juvenile bats into the population each year contributed to the circulation and maintenance of KKV. Experimental infections of free-tailed bats were also conducted to study the susceptibility and pathology of KKV in free-tailed bat hosts.

## 2. Materials and Methods

A description of the study area has been reported elsewhere [12]. This research was conducted in 1973–1974.

### 2.1. Study Site

The Kaeng Khoi cave is located 180 m above the valley floor on the steep western face of Khao Lorn Phat, a mountain which forms part of the eastern border of the valley (Figure S1). The cave has two exits; the main exit faces west, while the other exit is a hole in the ceiling at the rear of the large central room in the cave. The cave is divided into six rooms by limestone abutments. Five rooms on the periphery are connected to the large central room at different levels (Figure S2) by means of large, walkable passageways. The floor dimensions, levels of inter-room connections, names and code numbers of the cave are given in Figure S2. Rooms 1 and 2 were the areas utilized in this study. These rooms were inhabited all year by large bat roosts and functioned as maternity rooms. Temperatures in these rooms, as measured by 7-day hygro-thermographs during January–April 1970, remained essentially constant (26.9 ± 1.4 °C). The relative humidity in the cave varied from 50 to 94% during January–April 1970. The wrinkle-lipped free-tailed bat *(Chaerephon plicatus)* was the most numerous of the vertebrates with a population approaching one million (see Results). The population of tomb bats *(Taphozous theobaldi)* was much lower and was estimated to be less than 100,000 individuals.

### 2.2. Estimates of the Bat Population

Cave-dwelling colonial free-tailed bats leave on foraging flights daily at around sunset (Figure S3). The first activities of the exodus are initiated 15 to 30 min earlier in the cave and take the form of a progressively increasing number of bats flying clockwise, high in the main room. This circular holding formation gradually increases in size until the bats begin to leave the cave in a steady stream. An attempt was therefore made to estimate the free-tailed bat population by timed photographic series of the exodus (Figure S4). A motor-driven Nikon F2 camera (Nippon Kogaku K.K., Japan) with a 50 mm (F 1.4) lens and a Honeywell Strobonar Pressmaster 810 were used. The camera was handheld 9.1 m directly below the exodus and at a distance of 9 m from the entrance of the cave. The field of exposure of the camera at 9.1 m was 26. 25 m^2^ (6.25 × 4.2 m^2^ exposure plane), with a depth of field of approximately 7.5 m to infinity. The distance of 9.l m between the camera and the bats in the exodus remained essentially constant because of the structural limitation of the cave entrance. The cross-section of the exodus at the exposure plane varied in dimensions (1–3 m) as the number of bats in the exodus increased and decreased. Exposures (Tri-X film at ASA1000 film speed) were started when the first bats left the cave and were made at 30 s intervals for the first 7–10 min and thereafter at one- or five-min intervals. Each exposure of a photographic exodus series (Figures S3 and S4) was enlarged to 20 × 30 cm prints (Figure S3) and the number of bats on these prints were counted. With the aid of a stopwatch, it was determined that the bats crossed through the 6.25 m exposure field in one second, or at a speed of 22.5 km per hour. Since the number of bats represented in one exposure was an approximation of the number of bats that left the cave in one second, it was a simple task to estimate the number of bats leaving the cave over tiered intervals. The number of bats leaving the cave per time interval was, therefore, calculated by averaging the number of bats in two consecutive exposures and multiplying this average by the number of seconds between the two exposures.

### 2.3. Collection of Specimens

Normal adult and newborn bats were caught by hand in the mid-morning hours (06:00–08:00) from rooms 1 and 2 (Figure S2). They were then bled, sexed and individually identified. Moribund and dead bats were collected from the floors of rooms 1, 2, and 3.

Blood from adult bats was drawn by cardiac puncture with 2.5 mL disposable syringes. To obtain blood from newborn bats, a skin pouch was made around the axillary artery. The artery was then cut and the blood collected in the pouch was withdrawn by syringe. Whole blood from bats was diluted 1:2 (sera 1:4) at the time of bleeding by drawing the blood into syringes containing appropriate volumes (0.5 mL for adult and 0.25 mL for newborn) of sterile tissue culture growth medium 199 (GM). Oral swabs from bats were collected by massaging the oral cavity with a cotton swab. Urine was collected from handheld urinating bats with Pasteur pipettes. Oral swabs and urine samples were diluted in l.0 mL GM. All specimens were stored on dry ice at the field station until their transfer to the main laboratory, where the sera were stored at −20 °C. The blood clots, oral swabs, urine samples and animal bodies were stored at −60 °C.

### 2.4. Virus Isolation and Identification

Vero cell cultures (African green monkey kidney) and/or Swiss mice obtained from the Southeast Asian Treaty Organization (SEATO) mouse colony were used in virus isolation attempts. Vero cells were propagated in Blake bottles. Monolayers were removed with typsin-versene, resuspended to a final concentration of 10^5^ cells/mL in GM. GM contains medium 199 with 1.4 g Na HCO_3_ per liter, and was supplemented with 10% heat-inactivated fetal calf serum, 100 units penicillin/mL and 10 μg streptomycin/mL. Tissue culture tubes and 1 oz prescription bottles were seeded with 0.9 mL and 4 mL of cell suspensions, respectively, and incubated for four days at 37 °C prior to inoculation.

Individual tissues were triturated in sterile, 7 mL Ten Broeck tissue grinders containing 2 mL GM. The resultant suspension was centrifuged at 9750 *g* for 30 min at 4 °C. The supernatant was withdrawn and divided into two parts; one portion was frozen at −60 °C for re-isolation attempts, and the other one was inoculated intracerebrally (i.c.) into a litter of newborn Swiss mice (0.02 mL per mouse) and/or into 3 tubes of Vero cells (0.1 mL/tube). Urine, oral swabs and tissues from moribund bats were inoculated into Vero cell cultures only. Drained cell culture tubes were inoculated with 0.l mL of supernatant and incubated for l h at 37 °C. After adsorption, maintenance medium (1 mL) was added. Maintenance medium (MM) was GM supplemented with 5% fetal bovine serum. The inoculated cultures were incubated at 37 °C, and were examined for virus-induced cytopathic effect (CPE) over a period of 14 days. Supernatants from tubes showing CPE were sub-passaged. Maintenance medium was changed every third day. Inoculated mice were observed for 15 days. Brains of those found sick or dead were harvested and sub-passaged by intracerebral inoculation of mice. Virus isolates were identified by neutralization (N) tests, using Kaeng Khoi hyperimmune rabbit serum or mouse ascitic fluid. These tests were done with constant serum and varying virus dilutions.

### 2.5. Serologic Tests

Neutralization tests for antibodies against KKV were performed by plaque reduction or in tube cultures using Vero cell monolayers. Sera were inactivated at 56 °C for 30 min and diluted with GM. Virus was diluted to give an effective virus dose of 100 TCID 50 (50% tissue culture infectious dose) or 50–180 p.f.u. (plaque forming units). A 0.4 mL volume of each serum dilution was mixed with an equal volume of an appropriate virus dilution. The serum–virus mixture was incubated for one hour at 37 °C. Following incubation, two drained 1 oz. prescription bottles were inoculated, each, with 0.3 mL of serum-virus mixture. After adsorption at 37 °C for 1 h, 8–10 mL of overlay medium was added to each 1 oz. bottle. The overlay medium consisted of equal volumes of sterile 2% Ionagar No.2 (Oxoid Division, Oxo Ltd., London, England [13] in distilled water and of fluid medium containing the following pre-sterilized components: 51. 3 mL heat-inactivated (at S6 °C for 30 min) fetal calf serum; 1.2 mL of a 5% yeast extract; 3 mL of a 10% lactalbumin hydrolysate; 5.3 mL NaHCO_3_ solution (7.5% stock); 1.8 mL 100× penicillin-G and streptomycin; and 3 mL neutral red (1: 1000). Drained tubes were inoculated with 0.1 mL of serum-virus mixture. After absorption at 37 °C for 1 h, 1 mL of MM was added to each tube. Bottles and tubes were incubated at 37 °C and observed, respectively, for plaques and CPE on day five. A plaque reduction of 80% or more and no CPE in tubes were the criterion for a positive test.

### 2.6. Bats

The free-tailed bats used for experimental infection were collected from the Kaeng Khoi cave and were caged individually in cardboard boxes (15 *×* 15 *×* l5 cm^3^) with an inserted hanging screen. Bats were hand fed by a 2.5 cc syringe. A diet with the following ingredients was prepared in a Waring blender with a small amount of tap water: hardboiled egg yolk, cream cheese, and banana, each one part by weight, and ants, substituted for meal-worms, one half part by weight. The diet was fed once daily, approximately 2 cc per day.

### 2.7. Experimental Infection

A single pool of the prototype KKV strain, S-19-B, originally isolated from the brain of a dead free-tailed bat in 1969 [1,12], was used for all inoculations. The virus stock was prepared by harvest of Vero cell cultures showing CPE after inoculation with the ninth suckling mouse brain (SMB) passage of S-19-B virus. One hundred and thirty-six bats were initially infected, divided into multiple experimental groups (Table 1). Pre- and post-inoculation sera were tested for presence of neutralizing antibodies by plaque reduction test. Blood, tissues, oral swabs and urine samples from infected bats were tested for presence of KKV by inoculation into Vero cell tube cultures. Livers from some infected and noninfected bats were harvested for histopathology.

One litter of suckling mice was inoculated i.c. with 2 × 10^2^ SMLD_50_ of KKV. Inoculated mice were observed for illness and livers were harvested from moribund mice for histopathology.

## 3. Results

### 3.1. Estimates of the Free-Tailed Bat Population

The population size of free-tailed bats in Kaeng Khoi cave was estimated by recording the number of bats leaving the cave by a timed photographic series. Image-counting methods are a commonly employed method to estimate the population size of large cave-dwelling bat populations [14,15].

This method has four variables which could influence the population estimates: the exit speed of the bats may not be constant; the distance between the camera and the exodus may vary; there may be multiple bat species in the exodus; and, finally, all the bats may not leave the cave during the exodus. It was observed that the exiting speed and the exodus to camera distance did not change measurably during periods when the bat exit rate remained above 15 to 35 bats per second. At lower rates the free-tailed bats flew out of the cave sporadically, at different levels from the camera and at varying speeds. The tomb bat left the cave only after the free-tailed bat exit rate was below 15 to 35 bats per second. Generally, the adult bat roosts were empty in 30–40 min after the start of the exodus.

**Figure 1:**
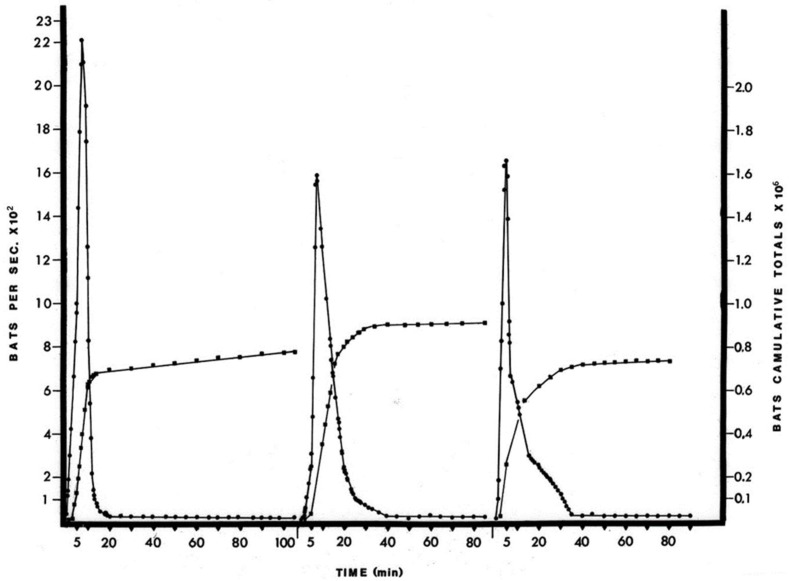
Pattern of wrinkle-lipped free-tailed bat exodus each month from Kaeng Khoi cave during April–June 1973.

In order to estimate the recruitment, a total of six estimates were made before, during and after the recruitment of juveniles into the foraging population. The population estimate varied between 700,000 and 900,000 in March–May, rose to 1.8 million in early June (when the bats birthed in April became volant), and declined to about 900,000 in late June (Figure S5). These values were derived from the cumulative totals (Figure 1 and Figure S5) when the bat exiting rate declined to 50 bats per second. This point was chosen because the cumulative totals, as well as the visual observation of the adult free-tailed bat roosts, indicated that the majority of bats had left on foraging flights by this time. The baby bats remained within the nurseries through the suckling period (mid-April to late May). Weaning and the initiation of foraging flights for the juvenile bats occurred during a short period (5 to 15 days) in late May and early June. Observations in the nurseries made after the adult bats had left the cave showed that the majority of juvenile bats were gathered in room 1, forming a layer one to three bats thick on the walls and floor. An inspection of the nurseries after the exodus on 7-Jun revealed that by this time all of the juvenile bats had joined the foraging population. The estimated population on this date was l.8 million bats, an approximate doubling in the number of foraging bats from the population values recorded during the suckling period.

The recorded population doubling at the time of weaning is what would be expected, since others [10] have shown that nursery colonies of free-tailed bats are composed of nearly 100% reproductively active females that give birth to one young each and that the mortality among the suckling bats is not high. The decline in total numbers of bats noted on June 21, fourteen days after the population doubling, may reflect the mature female bats leaving the cave for other roosting areas. This segregation of mothers from their young at the time of weaning has been shown to occur in other species of bats [16].

### 3.2. Transfer of Maternal Antibodies to Newborn Bats

Sera from bats of four age groups were tested for neutralizing antibodies to KKV. Sera from one-to-seven day old neonates and from reproductively active females were collected during the same time period (mid-April 1973) for a comparison of antibody titers and prevalence in the two groups. Most of the neonates had attached placentae. Further, the cohort born in mid-April was resampled in mid-May and in late June to obtain sera from 32–39 day old bats and 73–80 day old bats, respectively. Additionally, milk from ten seropositive lactating bats was obtained in late June to test for the presence of neutralizing antibodies to KKV.

The antibody prevalence in both the neonates and the reproductively active females was 84% and the distribution of antibody titers of positive sera was also comparable in these two groups (Table 2). These findings suggest that antibodies were readily passed from the mother to the newborn. The placenta of bats in the genus *Tadarida* is chorioallantoic [16] a placental type that is associated with the passive transfer of antibodies from mother to fetus. The antibody prevalence in 32–39 day old and 73–80 day old bats was 77% and 71%, respectively, and the geometric mean titer (GMT) of the 73–80 day old bats was about two-fold lower than that of the neonates. This decline in antibody prevalence and titers of an 80 day period appears to be less than what would be expected for trans-placentally transferred antibodies in a small mammal. In the laboratory mouse, the half-life of IgG is estimated to be 3–5 days [17]. The ten milk samples from the seropositive bats had no measurable neutralizing antibodies. Therefore, it is unlikely that the continued high antibody prevalence is due to transfer of antibodies in milk. The high prevalence of antibodies is also unlikely to be due to primary systemic infections, since the GMT declined with time.

### 3.3. Antibody Prevalence in Adult Free-Tailed Bats at Different Times

Sera from a total of 253 adult bats collected at different times of the year in 1973 were tested for prevalence and titers of neutralizing antibodies to KKV. The results are given in Table 3. The antibody prevalence varied between 91–29% from March through September. The variation in antibody prevalence observed is difficult to interpret without knowledge of the age distribution of the bats sampled and the age-specific KKV infection rates. The low antibody prevalence may reflect the presence in this population of juvenile bats (born in mid-April), most of whom have lost their maternal immunity. Tests for neutralizing antibodies to KKV were performed at a single 1:10 dilution of the sera.

### 3.4. Virus Isolation from Dead Freetailed Bats

In the earlier study [1] Kaeng Khoi virus was isolated from dead bats in each month from February through September 1969. In the present study, attempts were made to isolate virus from dead, moribund and healthy bats to obtain information on how KKV might be disseminated in nature (Table 4 and Table 5). Tissues of 20 dead bats collected in rooms 1, 2, and 3 on 5-Mar 1973, were examined for virus by mouse inoculation test. Virus was recovered from pooled tissues of 15 (75%) of the 20 dead bats (Table 4). The frequency of isolation from individual tissues of the virus-positive dead bats was as follows: brain 13/18; salivary gland 9/19; heart 12/19; and kidney 9/20.

### 3.5. Isolation from Moribund Bats

Tissues were collected from 24 moribund bats in rooms 1, 2, and 3 during January, February and March 1974 and examined for virus by inoculation of Vero cells. Virus was recovered from pooled tissue suspensions of 12 (50%) moribund bats (Table 4). Kaeng Khoi virus was found with a high frequency in oral swabs, urine, and blood of moribund bats (Table 5). Titration of KKV from the various specimens from morbid bats (Table 6) showed that considerable quantities of virus were present in all specimens examined; highest viral titers were noted in liver and lung suspensions. Blood and oral swabs of each of the four bats were positive for infectious KKV; viral titers in blood were higher than those in the oral secretions. Because of the high titered viremia in these bats, it is difficult to interpret the significance of tissue titers in terms of viral multiplication in the different organs. However, the much higher titers in the liver and lung as compared to those in the blood suggest that the virus multiplied in these organs.

**Figure 2:**
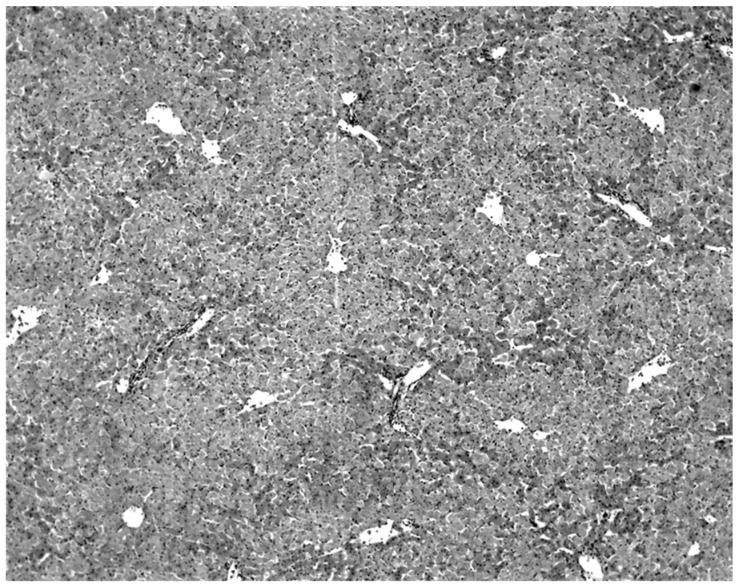
Massive liver cell destruction in a moribund wrinkle-lipped free-tailed bat naturally infected with KKV.

A histological examination of the tissues of morbid bats showed that viral infection was associated with a massive destruction of the liver parenchymal cells (Figure 2). A grossly-enlarged liver in moribund bats infected with KKV was appreciated, compared with the liver of KKV-negative bats (Figures S6–S8).

### 3.6. Isolation Attempts from Apparently Healthy Bats

Tissues of a total of 324 bats, including 68 bats less than nine weeks old, were collected over a seven month period and tested for KKV by inoculation into both in Vero cell cultures and suckling mice (Table 7). No isolations were made.

Virus isolation from dead and morbid bats suggests that KKV produced a marked systemic infection and some mortality, and that KKV could disseminate from an infected bat through saliva, urine and/or by a hematophagous arthropod. The lack of isolation from the apparently healthy bats suggests that the virus typically produces an acute, rather than a chronic infection.

### 3.7. Pathological Lesions of Kaeng Khoi Virus

The pathological lesions produced in bats infected with KKV were studied by three approaches. Histopathological examination was performed on (i) tissues from naturally infected morbid bats from Kaeng Khoi cave; (ii) livers from experimentally infected bats; and (iii) livers from experimentally infected suckling mice. The tissues were fixed in buffered formalin, embedded in paraffin, sectioned, and stained with hematoxylin and eosin for histopathological examination.

### 3.8. Lesions in Morbid Bats

Twelve of 24 morbid bats had naturally acquired KKV infection. The highest KKV titers were consistently found in the liver and the lung (Table 6). Of these, tissues were examined from 11 virus-positive and 5 virus-negative bats. The most characteristic lesion associated with KKV infection was in the liver (Table 8, Figure 2). Macroscopically, the liver was enlarged and was a dark reddish-brown in color (Figures S6–S8). Microscopic examination revealed an acute massive destruction of the liver parenchymal cells (Figure 2).

**Figure 3:**
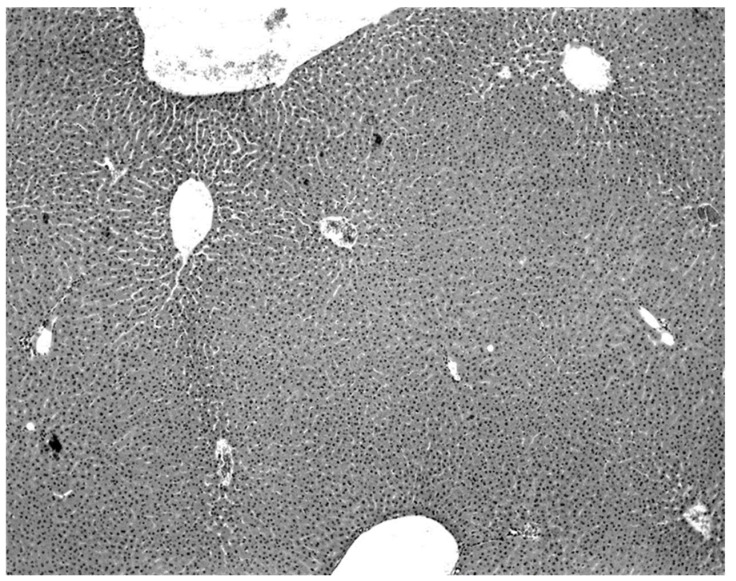
Normal liver of a KKV-negative naturally moribund free-tailed bat.

A consistent feature of the pathological process was the absence of an inflammatory infiltrate. Intranuclear and intracytoplasmic inclusion bodies were not seen. All hepatic lesions observed were well-advanced and were probably responsible for the mortality and morbidity in the infected bats. Livers of the virus-negative morbid bats were essentially normal (Figure 3).

Lung pathology was observed regularly in KKV-positive and KKV-negative bats. These lesions varied from a mild focal hemorrhage to a severe acute interstitial pneumonia. Hyalinization of the pulmonary vascular walls was observed in ten of the 16 bats examined, and was severe in two cases. Interstitial pneumonia was more common in the virus-infected bats (Table 8) than in virus-negative bats.

### 3.9. Laboratory Infection of the Free-Tailed Bat

The massive liver destruction in morbid bats naturally infected with KKV and the high titer of virus in the livers indicated that KKV produced an acute hepatitis in wrinkle-lipped free-tailed bats. An attempt was made to confirm this relationship by experimental infection of normal free-tailed bats acquired from Kaeng Khoi cave.

Data on 35 experimentally infected bats are summarized in Table 9. Naturally acquired immunity to KKV protected against KKV challenge; none of the eleven bats which had antibodies to KKV at the time of challenge exhibited viremia or virus in tissues on days 2 to 5 after i.c. or i.p. inoculation of high doses of Kaeng Khoi virus. On the other hand, nonimmune bats inoculated i.c. with high or low doses of virus regularly developed viremia and had virus in their tissues by days 2 to 5. The virus titers in blood and tissues exceeded 10^4.0^·TCID_50_ (per ml of blood or gram of tissue).

Nonimmune bats appeared to be more susceptible to i.c. than to i.p. inoculation. In two cases, bats which were viremic on days 2 and 3, had developed antibodies by day 7; on day 7, the tissues had over 10^4^ TCID_50_ of virus per gram, while the blood had no detectable virus. Of the five bats inoculated i. c. with 2 × 10^2^ SMLD_50_, livers from four were harvested for virus titer on days 4–6 post inoculation. All four livers were positive for virus and contained 6.2, 6.3, 7.2 and greater than 8.1 log_10_ virus per gram of liver.

**Figure 4:**
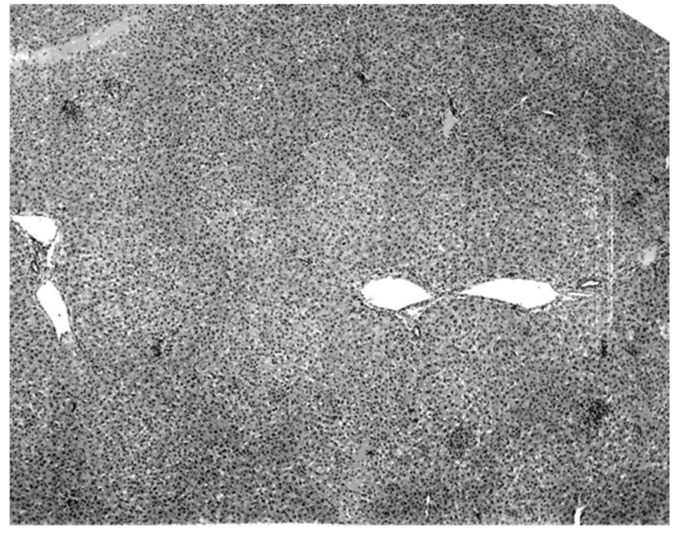
Acute multi-focal hepatitis observed in wrinkle-lipped free-tailed bats experimentally-infected with KKV.

**Figure 5:**
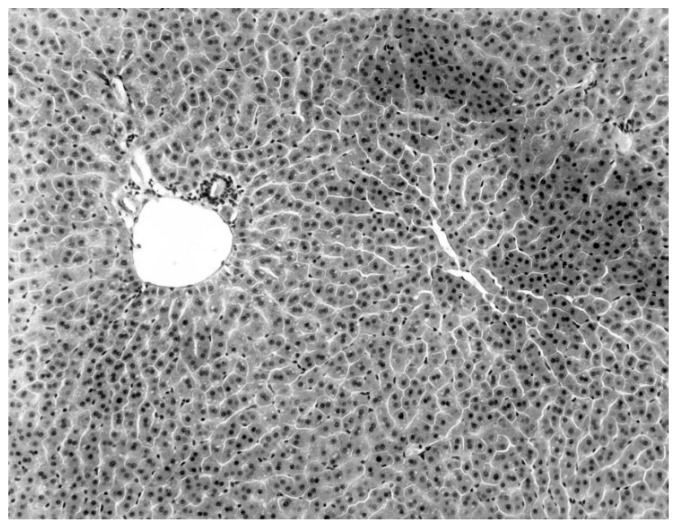
Liver section of a wrinkle-lipped free-tailed bat experimentally infected with KKV and harvested prior to day 5 post-infection, prior to when parenchymal cell destruction started occurring.

Pathological lesions characterized as acute multifocal hepatitis (Figure 4) were observed in all four infected experimental bats harvested on day 7 (Table 9). Of the three KKV-infected bats harvested on day 5 or before, two had essentially normal livers (Figure 5) and one, harvested on day 5, had a mild focal hepatitis. A cellular infiltrate was observed focally in and around all the liver lesions observed.

These data suggest that KKV is hepato-trophic for bats and that naturally acquired immunity protects the immune bats against a second systemic infection.

### 3.10. Laboratory Infection of Suckling Mice

A litter of six suckling mice inoculated with 2 × 10^2^ SMLD_50_ i.c. were harvested when moribund, 5–7 days post inoculation. All manifested a massive liver cell destruction similar to that observed in the virus-positive morbid bats (data not shown). The two control mice inoculated i.c. with virus diluent exhibited unremarkable livers.

## 4. Discussion

Several important components of the epidemiology of KKV infection of wrinkle-lipped free-tailed bat inhabiting Kaeng Khoi cave were studied during 1973–1974. The data presented support the natural circulation of KKV in free-tailed bats at a high prevalence, driven by the integration of immunologically-naïve juvenile bats into the population after maternal antibody protection waned. Importantly, infection of these bats with KKV did not appear to be benign; isolation of KKV was only from moribund or dead bats, and experimentally-infected bats developed pathogenic lesions in the liver and lungs. Virus isolations of KKV were also only made from dead wrinkle-lipped free-tailed bats in Cambodia, with no isolations from apparently healthy bats [7].

### 4.1. Recruitment of Susceptible Bats

The April birthing period was estimated to be about 900,000 bats or an approximate doubling of the cave population. Although the accuracy of the estimates is difficult to assess without knowing the true population values, the method did record a doubling of the population in June, when these juvenile bats would have become volant (Figure S5). Alternative methods [10,18] for the estimation of large bat populations, i.e., by roost density, exodus trapping and recapture ratio, are problematic because they disrupt either the exodus flow or the roosting bats themselves. The photographic procedure simply records the natural exodus flight, but limitations include not being able to accurately differentiate bat species or age from the photographs. The free-tailed bat population returned to previous levels within two weeks of the initial June foraging flights (Figure S5). This increase in numbers of bats and the subsequent decrease in number of bats in the cave population represents recruitment through birth and then the dispersal of bats over a wide area and is probably representative over the species home range.

Serological results of the sampled newborns showed that 84% (approximately 700,000 of 900,000 recruited newborns) of pups had neutralizing antibodies to KKV (Table 2). These antibodies were likely of maternal origin, as this antibody prevalence matched that of the lactating female bats and was detected within days of birth (Table 2). The sampled newborn bats were too young to have acquired antibodies from an outside infection, and, in addition, attempts to isolate virus from them were consistently negative. These antibodies were most likely transmitted via the placenta, since these bats have a hemochorial placenta which permits antibody passage [16]. Neutralizing antibodies were not found in the milk of ten seropositive lactating females (data not shown).

The high antibody prevalence rate in the newborn bats may be expected to protect the seropositive young from systemic KKV infection. The length of time maternal antibodies would protect the newborns would depend upon the half-life of the IgG immunoglobulin of bats, the initial titer of the antibodies and perhaps the dosage of KKV that the bats are exposed to in the cave. It is not known at what time after birth the bat would become susceptible to KKV infection. The cohort of baby bats born in mid-April maintained a high antibody prevalence of 77% and 71% at 32–39 and 73–80 days of age, respectively (Table 2), but with some decrease in titer. This attrition suggests that maternal antibodies were still present at the time of weaning in late May and early June, about 6–7 weeks after birth, and that the majority of bats in the mid-April birth cohort would probably become susceptible to KKV over the next few months (July, August and September). Thus, of the 900,000 bats born in April, over 100,000 seronegative individuals were susceptible to KKV infection at birth and the remainder would provide the susceptible population over a period of several months. Transfer of maternal-derived (MDA) antibodies from female bats to pups, and the persistence of these antibodies has been documented in several other disease systems. Epstein et al. monitored the half-life and maintenance of MDA in both captive *Pteropus hypomelanus* bats vaccinated against canine distemper virus (CDV), and Hendra virus antibodies in wild-caught *P. alecto* bats [19]. In both cases, transferred maternal immunity lasted approximately 7.5–8.5 months, with an antibody half-life of 96 days for CDV and 52 days for Hendra. Maternal antibody protection of African straw-colored fruit bats (*Eidolon helvum*) exposed to Lagos bat virus or African henipa-virus was about six months, with a low point in seroprevalence at 11–12 months [20]. The waning of MDA in large bat populations with synchronous birthing is known to contribute to the dynamics of viral transmission and maintenance, as pulses of immunologically-naïve individuals enter the population at once [20,21,22,23]. Wrinkle-lipped free-tailed bats have two birthing periods per year, an ecological attribute important to the maintenance of filoviruses [21,23]. Marburg virus infection rates peaked in older juvenile Egyptian rousette bats in Uganda during the birthing period when the next cohort was born, presumably due to the waning of MDA in those older juveniles [23].

The antibody prevalence rates of the adult bats sampled from March 1973 through September 1973 are difficult to interpret because reliable methods are not available for the estimation of the age of the free-tailed bats older than about four months. Further, the movement patterns of these bats among caves and other roosting areas are unknown, so it is difficult to appreciate whether or not the samples taken at different times of year represent the same group of individuals. However, it is likely that the presence of the reproductively active females accounted for the high antibody prevalence observed in March, April and May and that the low antibody prevalence rates found in August and September reflect the loss of maternal immunity by the cohort of bats born in mid-April (Table 3).

### 4.2. Kaeng Khoi Virus Infection in the Bat

Of the 136 bats initially included in the three experimental groups (Table 1), 35 bats were studied sufficiently and are discussed in the “Results” Section. The difficulties in conducting the experiment were as follows: (i) the histories and physical condition of the bats were unknown; (ii) many of the bats had naturally acquired antibodies—the bats were acquired from a KKV endemic region and the immune status of some bats was not known at the time of inoculation; (iii) there was considerable nonspecific mortality due to handling and captivity. In retrospect, most of these difficulties could have been avoided, if the sera of the captured bats had been first examined for antibodies and the infection attempted in antibody-negative bats which had survived the first week of captivity. Still, pathology data are presented in terms of the immunological status of the bats, from which some differences were observed (Table 9). The presence of pre-existing antibodies from KKV exposure did appear to be protective against subsequent infection. By comparison, infection-induced antibody titers decayed much more slowly than MDA, and were highly variable in African straw-colored fruit bats [22].

The only characteristic lesion found in naturally infected moribund bats was an acute massive destruction of the liver parenchymal cells. All of the moribund bats found positive for KKV had hepatic lesions which were well advanced and were probably responsible for the mortality and morbidity in the infected bat (Figure 2). Livers of KKV-negative morbid bats were essentially normal (Figure 3). Suckling mice inoculated with KKV also died with a massive destruction of their liver parenchymal cells (data not shown). Furthermore, experimental infection produced an acute hepatitis in the free-tailed bat (Figure 4). These virus isolation and pathology data show that KKV is hepatotropic and seems to produce hepatitis in the free-tailed bat that may lead to the death (Table 4, Table 6 and Table 9; Figure 2 and Figure 4). However, a major limitation of this analysis is that immunohistochemical localization of viral antigen in the affected tissues was not performed to associate the pathology directly with the presence of KKV in the same sample. Hepatitis is characterized by perivascular infiltrates as seen in Figure 2, but it is unclear what cell types are involved in this case. Viruses from multiple families within the order *Bunyavirales* characteristically produce liver destruction, including many viruses in the family *Peribunyaviridae*: Caraparu (group C) [24], Patois [25], Guama [26] Oropouche [27]; *Phleboviridae*: Rift Valley fever virus [28,29,30]; *Nairoviridae*: Crimean Congo hemorrhagic fever virus [29] and *Hantaviridae*: hemorrhagic fever with renal syndrome (hantaviruses) [29].

The high isolation rate of virus from sick and dead bats and the lack of isolation from healthy bats, together with the severe hepatic lesions in the sick bats, strongly indicate that KKV infection produces mortality and that it may be responsible for as many as 50–75% of the deaths occurring in the cave (Table 4). These observations were made during March 1973 and January–April 1974, but they are probably representative of what occurs throughout the year. February–April also represents the dry season, in which food is less available and energetic stresses were noted among bats in this species in Cambodia [11]. The relationship between reproductive phenology, virus shedding, and energetic reserves over the wet and dry seasons should be further investigated. Patterns and duration of viral shedding by bats over time are also not yet known.

In 1969 an attempt was made to estimate the number of bats dying in the cave. All dead bats found on the floor of the cave were collected three times a day, for one week of each month, for twelve months. The highest number of daily deaths for any one month did not exceed 30 bats, giving a maximal estimate of annual deaths in the cave of about 11,000, or between 1–2% of the population in the cave. This estimate seems to be very low, and suggests that a large majority of deaths are occurring outside the cave. However, the age structure and movement patterns of the bat population in the cave during this period of time were not known and, therefore, this estimate only indicates the number of deaths occurring in the cave and cannot be interpreted as a mortality rate or to indicate what proportion of this mortality outside the cave is due to KKV. The significance of mortality due to KKV on the bat population warrants further investigation.

Although there is no good basis to calculate the mortality rate in bats infected with KKV, the large numbers of bats with immunity and the high proportion of newborns with maternal immunity suggest that the mortality due to this infection may be lower than indicated by the isolation rates from dead and moribund bats.

## 5. Conclusions

This manuscript describes a series of field- and laboratory studies that collectively provide important, novel information regarding the viral ecology of KKV. Investigations into the population ecology and seasonal reproductive phenology of this bat population were highly informative to the circulation and maintenance of KKV. Importantly, bats appear to be susceptible to KKV, with significant liver pathology noted in dead and moribund bats infected with KKV. Specific key findings are summarized below:

1. The population of bats in the Kaeng Khoi cave was estimated by a timed photographic series of the evening exodus. The initial population of between 700,000 and 900,000 bats (between March and May 1973) nearly doubled in early June and declined to its previous level in late June. The increase in number was due to the recruitment of bats born in mid-April into the foraging population.
2. Neutralizing antibodies to KKV were probably transferred from mother to the newborn. The antibody prevalence in adult bats was high in March–May 1973 and low in August and September 1973.
3. Kaeng Khoi virus was isolated from 75% of dead, 50% of moribund and no isolations were made from healthy bats. In moribund bats, KKV was present in all tissues examined, with the highest titers in liver and lung tissue. Virus was routinely isolated from the blood, urine and saliva of infected bats.
4. The characteristic pathology in naturally infected moribund free-tailed bats was an acute massive destruction of liver parenchymal cells. Experimentally infected bats and suckling mice also exhibited liver lesions. Bats with naturally acquired antibodies resisted the Kaeng Khoi virus challenge.

## Figures and Tables

**Table 1:** Groups of bats experimentally inoculated, routes and doses, and specimens collected.

Group	Date (1973)	Inoculum		Specimens Collected
		Route *	Dose (SMLD_50_)	
1	23-Aug	i.p.	8 × 10^6^	Serum, days 2 and 3; tissues, day 7 or time of death
		i.c.	3 × 10^5^	
2	23-Sep	i.c.	2 × 10^2^	Serum, tissues, and oral swabs on days 1, 2, 4, 5
		i.n.	1 × 10^1^	
3	28-Sep	i.c.	4 × 10^1^	Serum and tissues on days 1, 2, 4, 5; urine daily for 5 days

* i.c = intracerebrally;- i.p. = intraperitoneally; i.n. = intranasally.

**Table 2:** Transfer of maternal antibodies: antibody prevalence and titers in different age groups of free-tailed bats, 1973. GMT = geometric mean titer.

Category ^1^	No. of Sera Examined	No. (%) Bats with Antibodies	No. (%) Positive Sera with Titers > 200	GMT
Reproductively active females	38	32 (84)	13 (41)	57
Neonates ^2^ (1–7 days old)	61	51 (84)	23 (45)	58
32–39 days old ^3^	31	24 (77)	3 (12)	40
73–80 days old ^4^	24	17 (71)	4 (23)	30

^1^ Collection times mothers and neonates 10 April 1973, 32–39 days old 12 May 1973, 73–80 days old 21 June 1973. ^2^ Forearm length 19.5 ± 1.6 mm, fetus of same date 18.1 ± 1.1. ^3^ Forearm length 42.6 ± 1.6 mm. ^4^ Forearm length 46.6 ± 0.05 mm, forearm length of reproductively active female is 45.9 ± 1.3 mm; hair coloration of these bats was light gray, and that of adults was brown.

**Table 3:** Frequency of neutralizing antibodies to KKV in adult free-tailed bats in different months of the year, 1973. * Not Done. GMT = geometric mean titer.

Date of Collection	No. Examined	No. (%) with Antibody	No. (%) Positive Sera with Titers > 200	GMT
17-Mar	58	53 (91)	10 (19)	30
10-Apr	63	42 (67)	19 (45)	63
12-May	13	9 (69)	1 (11)	-
22-Aug	66	19 (29)	0 (0)	19
23-Sep	53	21 (40)	ND *	-
Total	253			

**Table 4:** Frequency of virus isolations from dead, moribund, and healthy bats.

Category	Date	No. Examined	No. (%) Positive
Dead	Mar-73	20	15 (75)
Moribund	Jan–Mar 1974	24	12 (50)
Healthy	Feb–Aug 1973	393	0 (0)

**Table 5:** Frequency of virus isolations from blood, urine, and oral swabs from moribund bats.

Specimen	No. Processed	No. (%) Positive
Oral swab	11	10 (91)
urine	7	5 (71)
blood	10	9 (90)

**Table 6:** Distribution of KKV in tissues of naturally-infected moribund bats.

Bat No.	Tissues Examined							
	Brain ^1^	Liver ^1^	Kidney ^1^	Spleen ^1^	Salivary Gland	Lung ^1^	Blood ^2^	Oral Swab ^2^
S-0022	7.1	8.3	ND ^3^	ND	ND	ND	6.0	3.0
S-0023	Neg ^4^	8.5	5.0	Neg	4.7	9.1	5.0	4.0
S-0024	7.6	8.2	7.5	9.6	7.1	9.1	7.0	6.0
S-0025	7.0	9.5	6.1	6.1	7.4	8.1	7.0	6.0

^1^ Log_10_ pfu per gram of tissue. ^2^ Log_10_ pfu per mL of tissue. ^3^ not done. ^4^ negative.

**Table 7:** A summary of virus isolation attempts from tissues of adult and juvenile wrinkle-lipped free-tailed bats collected from Kaeng Khoi cave ^1^.

Collection ^2^ Dates 1973	Adults				Juveniles			
	Total No. Bats	No. Isolates/No. Specimens			Total No. Bats	No. Isolates/No. Specimens		
		Brain	Blood	Salivary Gland		Brain	Blood	Salivary Gland
13-Feb	49	0/36	0/48	ND	-	-	ND	-
17-Mar	54	0/39	0/50	0/25	-	-	ND	-
10-Apr	63	0/9	0/63	0/9	56	ND	0/56	ND
12-May	29	0/6	0/23	ND	5	ND	0/5	ND
21-Jun	-	-	-	-	7	0/7	-	-
July	-	-	-	-	-	-	ND	-
22-Aug	61	0/26	0/61	0/26	-	-	ND	-
Total	256	0/116	0/245	0/60	68	0/7	0/61	-

^1^ In March 1974, 69 oral swabs and 50 urine samples were taken from 69 adult bats; all were negative for KKV. Total number of bats processed was 325 (608 specimens). ^2^ Bats were collected at a time of evening exodus in February; at all other times they were collected in the cave by hand in the early mid-morning hours (06:00–07:30).

**Table 8:** Pathological lesions in tissues of virus-positive and virus-negative morbid bats.

Tissue Specimens	No. Examined	Pathological Lesions		
		KKV-Positive Bats	No. Examined	KKV-Negative Bats
Brain	11	unremarkable	5	unremarkable
Kidney	11	unremarkable	5	unremarkable
Salivary gland	2	unremarkable	3	unremarkable
Fetus	3	unremarkable	0	-
Spleen	3	2 unremarkable; 1 necrosis, lymphoid, acute diffuse, mild	1	unremarkable
Lymph node	3	1 unremarkable; 2 necrosis, lymphoid, acute mild diffuse, lymph node	0	
Lung	11	8 hyalinization of pulmonary vascular walls; 5 focal interstitial pneumonia, acute; 1 focal hemorrhage	5	2 hyalinization of pulmonary vascular walls, congestion; 1 focal hemorrhage; 1 hemorrhagic pneumonia, severe; 1 acute interstitial pneumonia, severe; 1 unremarkable
Liver	8	massive destruction of all livers	4	unremarkable

**Table 9:** Hepatitis and viral isolations from laboratory-infected free-tailed bats.

Route of Inoculation	Dose (SMLD_50_)	No. Inoculated	Virus in Blood and Tissues ^1^				Neutralizing Antibodies in Survivors ^2^	Severity of Hepatitis ^3^		
				Days Post Inoculation				Day of Harvest		
				1–2	3–5	6–7		2	5	7
Non-Immunes										
i.c.	3.2 × 10^5^	3	blood	2/3	3/3	0/2	2/2			2+
			tissue			2/2				2+
	2 × 10^2^	5	blood				1/1			
			tissue		3/3	1/2				
	4 × 10^1^	7	blood	0/3			7/7	0	0	
			tissue	1/3	2/4				1	
i.p.	8 × 10^6^	9	blood	2/8	4/9		4/7			
			tissue		2/2	0/3				2+
Immunes										
i.c.	4 × 10^1^ to 3.2 × 10^5^	9	blood	0/2	0/3					
			tissue	0/2	0/5					
i.p.	8 × 10^6^	2	blood	0/2	0/2					
			tissue							

^1^ The ratios are the number of virus-positive/number examined. ^2^ Number positive/number tested. ^3^ Severity of hepatitis scored as follows: 1 = acute focal hepatitis, 2 = acute multifocal hepatitis.

## Data Availability

All available data are provided in the manuscript and Supplementary Materials.

## Supplementary Materials

The following are available online at https://www.mdpi.com/article/10.3390/diseases9040073/s1, Figure S1. Location of Kaeng Khoi cave (centrally on this map) in the Chao Phraya delta area of Thailand. Figure S2. Map of the rooms within Kaeng Khoi cave. Figure S3. Wrinkle-lipped free-tailed bats (*Chaerephon plicatus*) exiting Kaeng Khoi cave at sunset. This photo represents one enlarged exposure used to count exiting bats (exposure 17 from 11 April 1973 exodus series). Figure S4. Photographic exposure series of bats exiting Kaeng Khoi caves. The population size of wrinkle-lipped free-tailed bats was estimated by analysis of still-frame photos of the evening exodus from the cave. Figure S5. Cumulative totals of bats exiting Kaeng Khoi cave by month and birthing period. Note the population spike in June. Figure S6. Two fresh bat livers one (Left and small) from a morbid Kaeng Khoi negative bat and the other (right and large) morbid Kaeng Khoi positive bat. Figure S7. Enlarged liver in a KKV-infected bat. Figure S8. Normal liver in a KKV-negative bat.
