# Supplementary material for: Viral Ecology and Natural Infection Dynamics of Kaeng Khoi Virus in Cave-Dwelling Wrinkle-Lipped Free-Tailed Bats (Chaerephon plicatus) in Thailand
Source: Diseases. 2021 Oct 15;9(4):73. doi: 10.3390/diseases9040073 (PMC8544448; doi:10.3390/diseases9040073)
Supplement: Supplementary file 1 [file diseases-09-00073-s001.zip › diseases-1375194-supplementary.pdf]

Kaeng Khoi virus ecology – Supplementary material

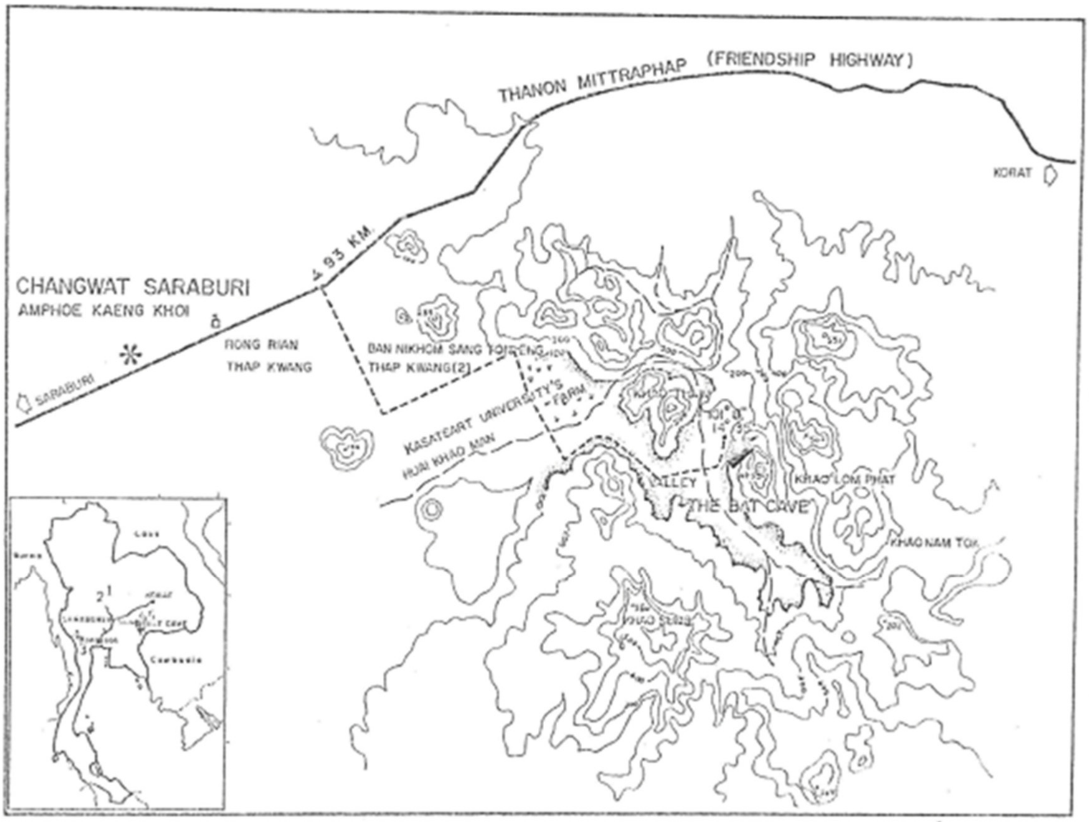

Figure S1. Location of Kaeng Khoi cave (centrally on this map) in the Chao Phraya delta area of Thailand.

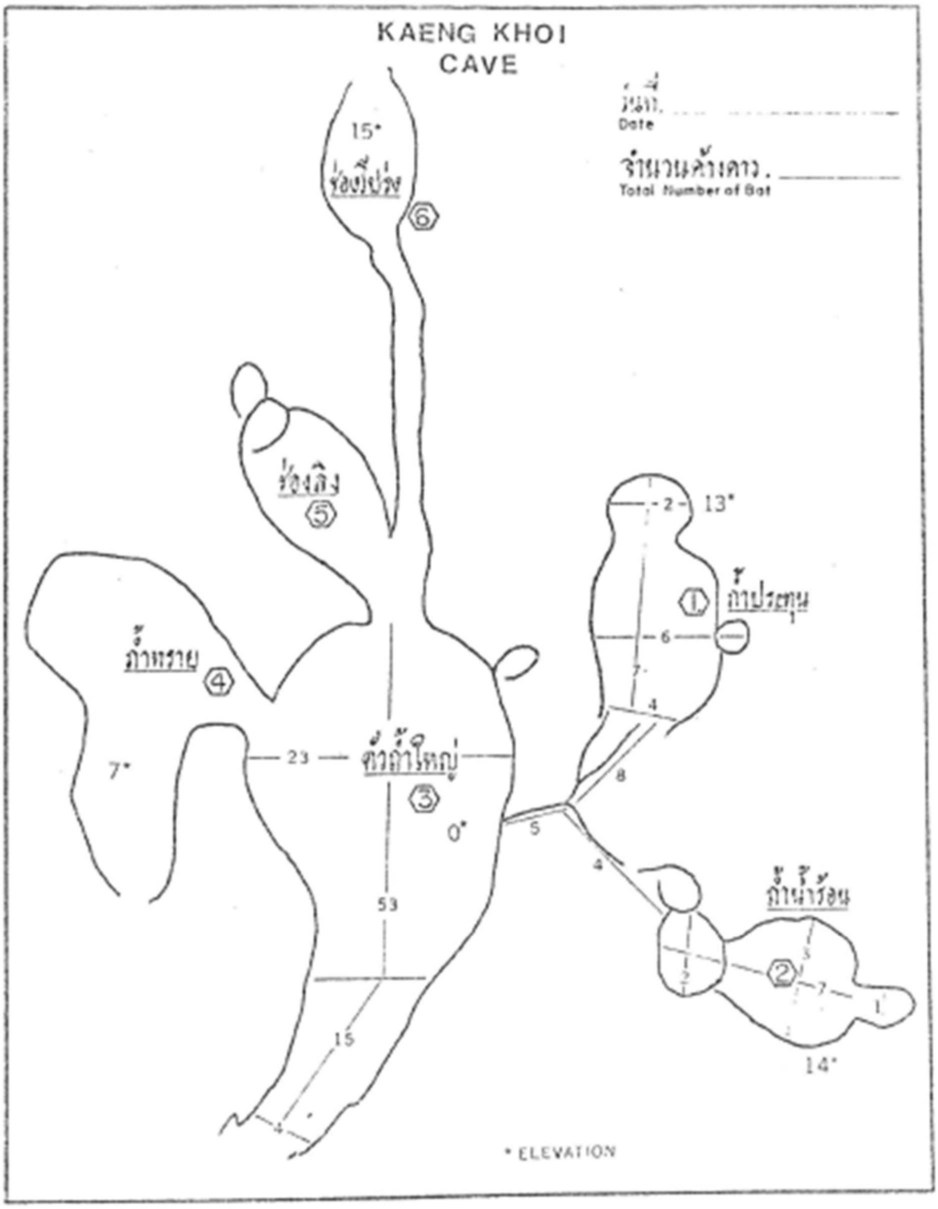

Figure S2. Map of the rooms within Kaeng Khoi cave.

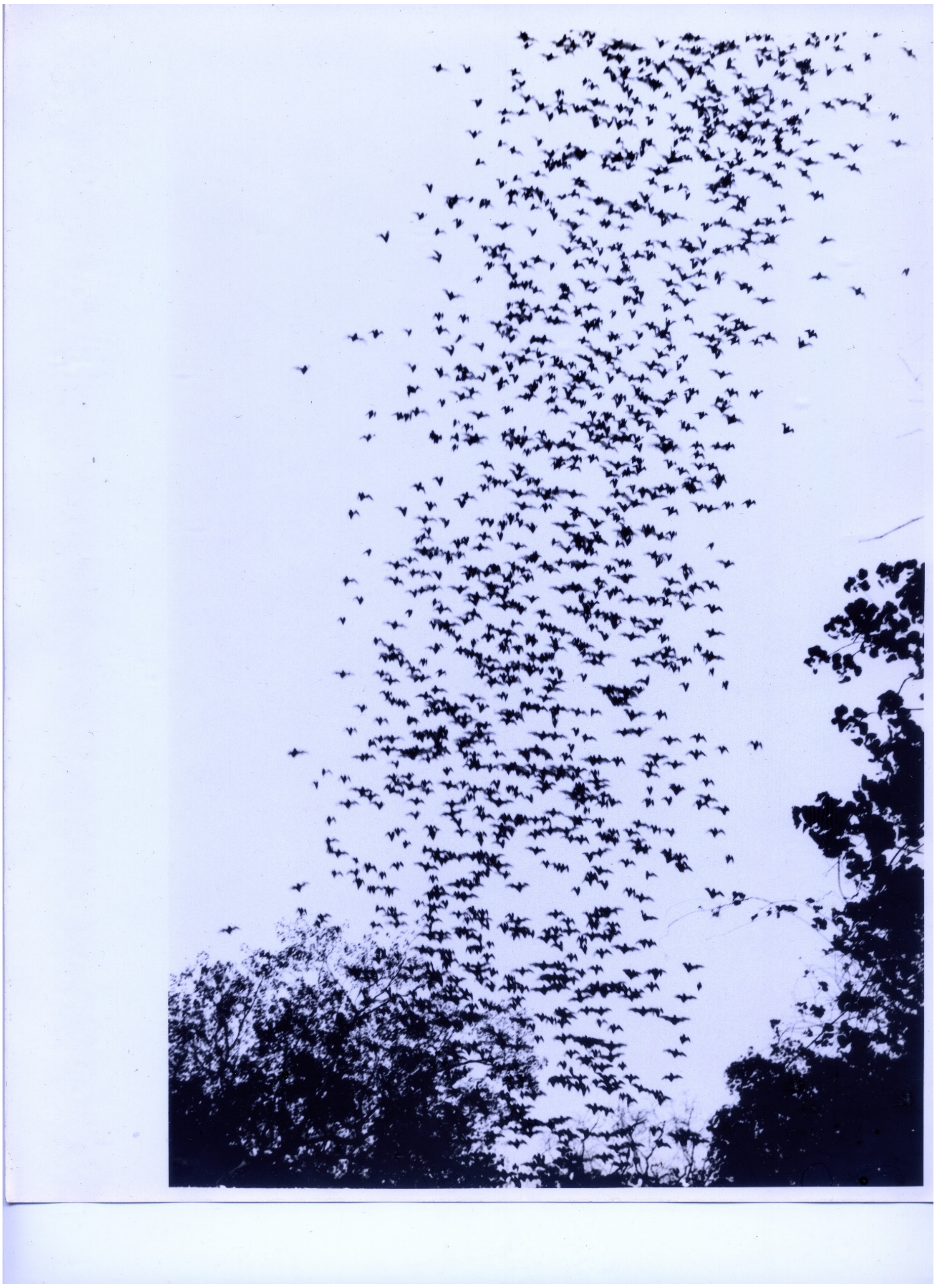

Figure S3. Wrinkle-lipped free-tailed bats (*Chaerephon plicatus*) exiting Kaeng Khoi cave at sunset. This photo represents one enlarged exposure used to count exiting bats (exposure 17 from 11 April 1973 exodus series). Photo by William Neill.

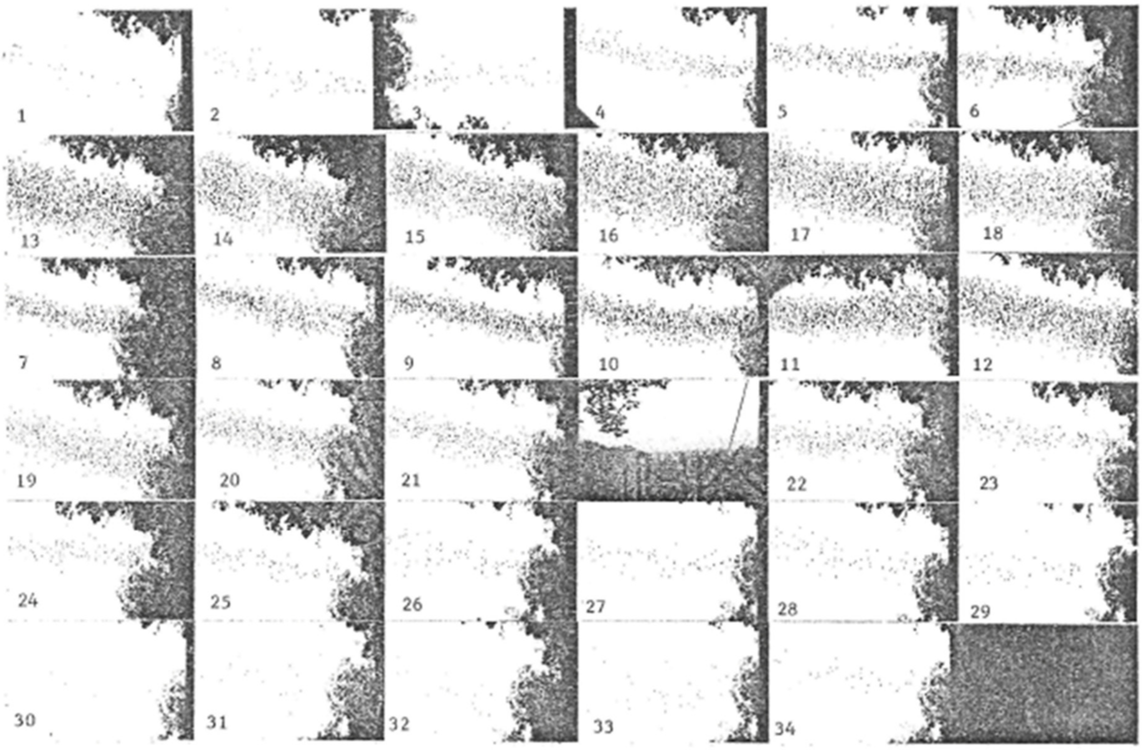

Figure S4. Photographic exposure series of bats exiting Kaeng Khoi caves. The population size of wrinkle-lipped free-tailed bats was estimated by analysis of still-frame photos of the evening exodus from the cave.

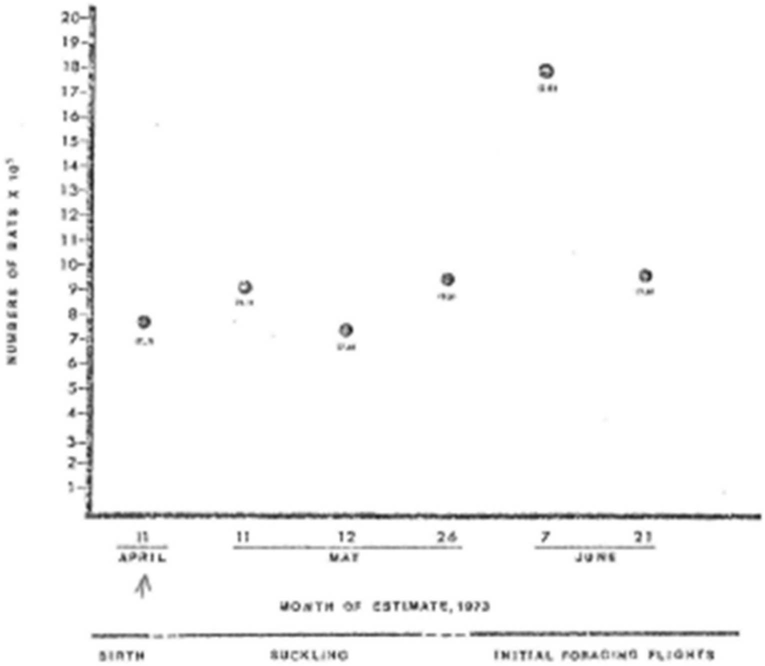

Figure S5. Cumulative totals of bats exiting Kaeng Khoi cave by month and birthing period. Note the population spike in June.

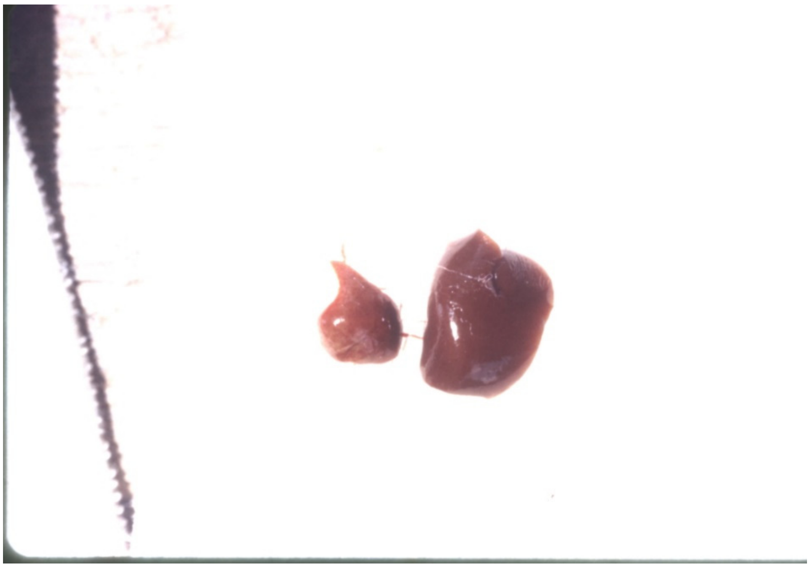

Figure S6. Two fresh bat livers one (Left and small) from a morbid Kaeng Khoi negative bat and the other (right and large) morbid Kaeng Khoi positive bat.

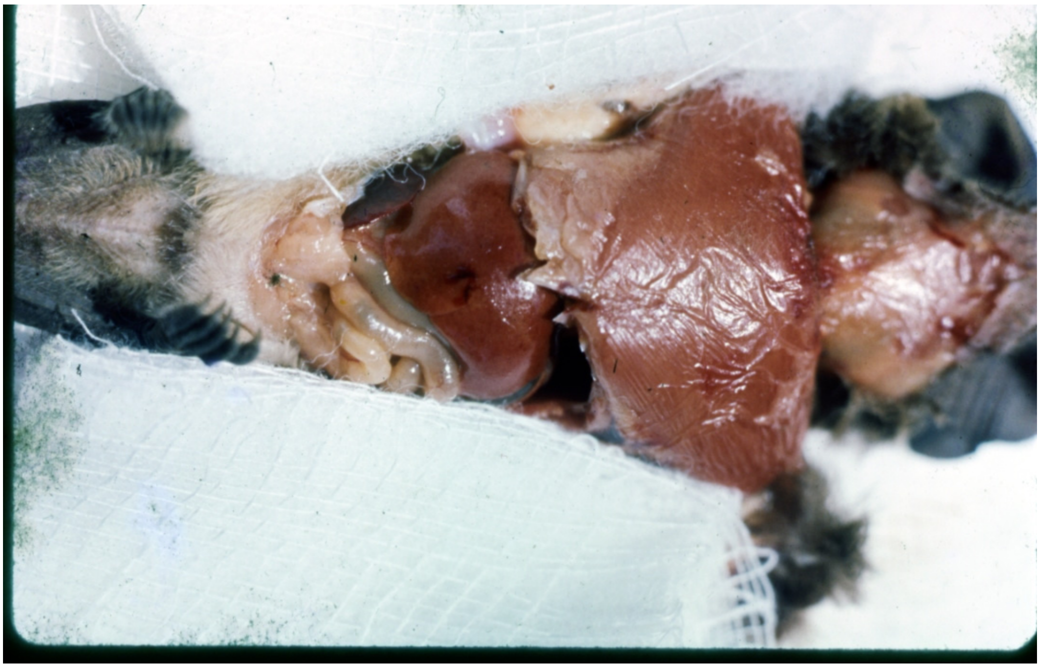

Figure S7. Morbid Kaeng Khoi positive bat showing enlarged liver.

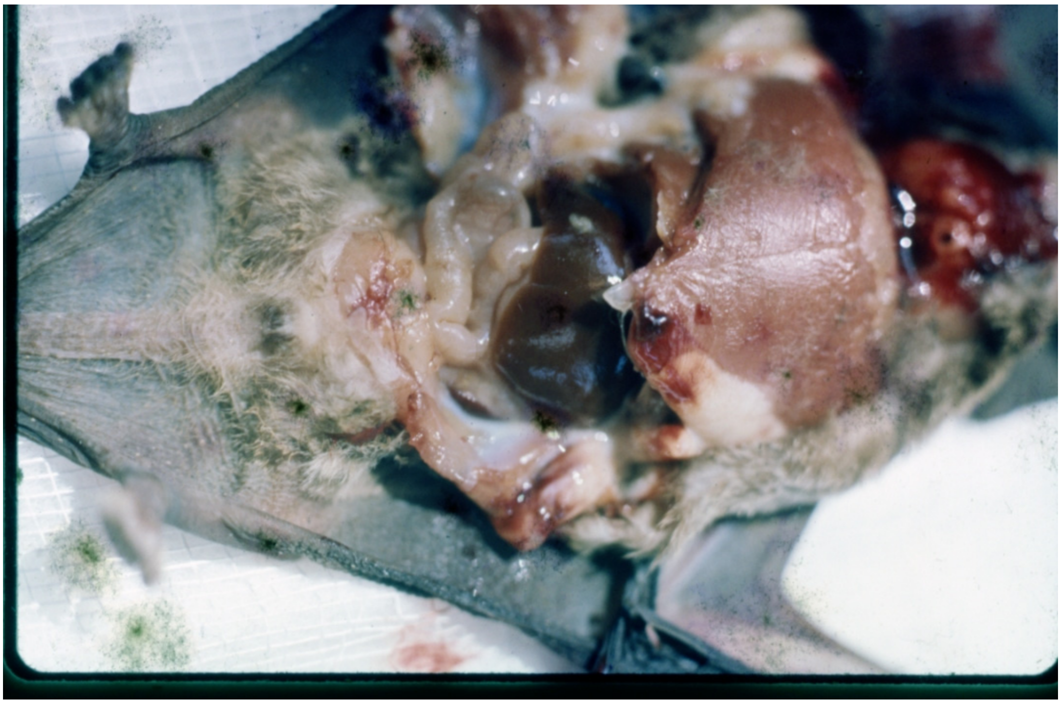

Figure S8. Morbid Kaeng Khoi negative "normal" liver.
